# Supplementary material for: Thyroid hormone promotes fetal neurogenesis
Source: JCI Insight. 2025 Sep 4;10(19):e194445. doi: 10.1172/jci.insight.194445 (PMC12513484; doi:10.1172/jci.insight.194445)
Supplement: Supplemental data [file jciinsight-10-194445-s128.pdf]

Supplemental figure legends

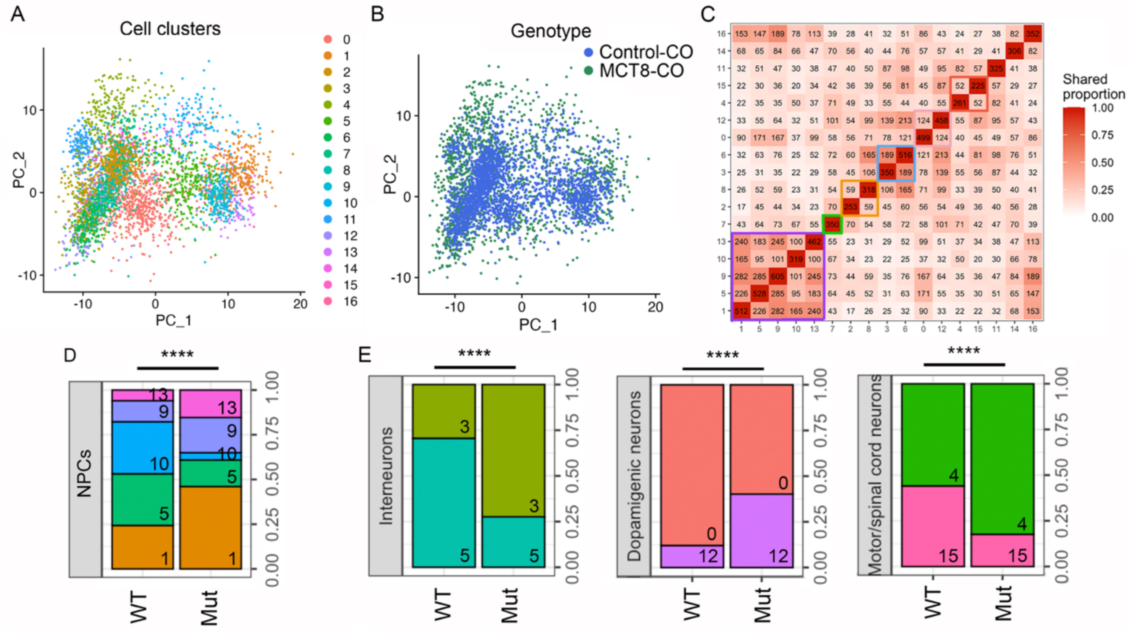

Supplemental Figure 1. A. PCA of the first two principal components (PC1 versus PC2) of D50 COs. **B**. Same as in **A**, except that the different genotypes are indicated. **C**. A plot of the number of conserved genes shared across the clusters. Coloured squares (purple, green, orange, blue, and red) indicate the cluster of cells for cells belonging to different cell types. **D**. Histograms of the relative number of cells in clusters of NPCs. The identification number of each cell cluster is indicated at the bottom right corner of each rectangle. **E**. Same as in **D** for the indicated type of neurons. The χ^2 test for multiple comparisons and pairwise cell proportions. ***P < 0.0001.

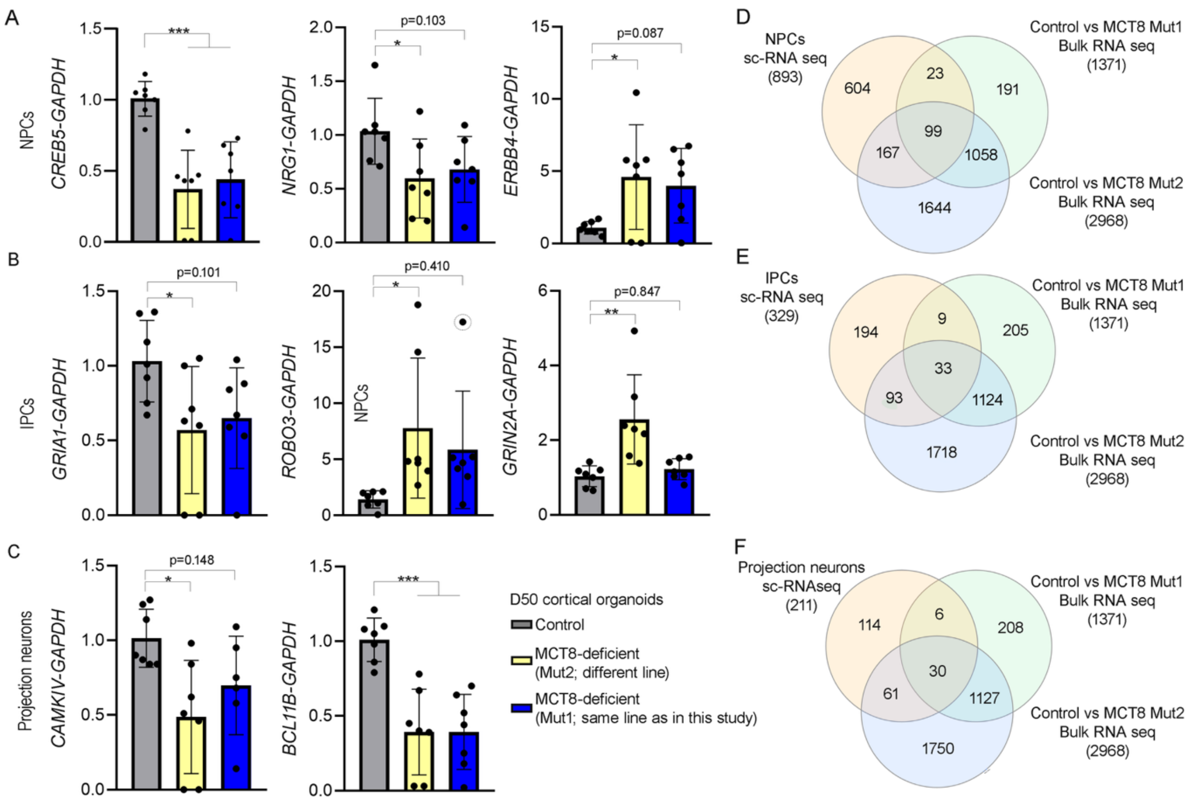

Supplemental Figure 2. A-C. mRNA expression levels of selected top differentially expressed genes identified in the single-cell RNA seq analysis of the cell clusters belonging to NPCs (**A**), IPCs (**B**), and projection neurons (**C**). Samples are from D50 control and MCT8-deficient cortical organoids obtained in previous studies (Salas-Lucia et al., 2024). Values are mean ± SD of 7 independent experiments; One-way ANOVA and the Tukey post hoc test were used for multiple comparisons; *P < 0.05, **P < 0.01, ***P< 0.001. **D-F**. Venn comparison of differentially expressed genes identified with single-cell RNA analysis of the cell clusters belonging to NPCs (**D**), IPCs (**E**), and projection neurons (**F**) in D50 COs and differentially expressed genes between D65 control vs. MCT8-deficient COs identified by bulk-RNA seq in Salas-Lucia et al., 2024; common differentially expressed genes were identified.

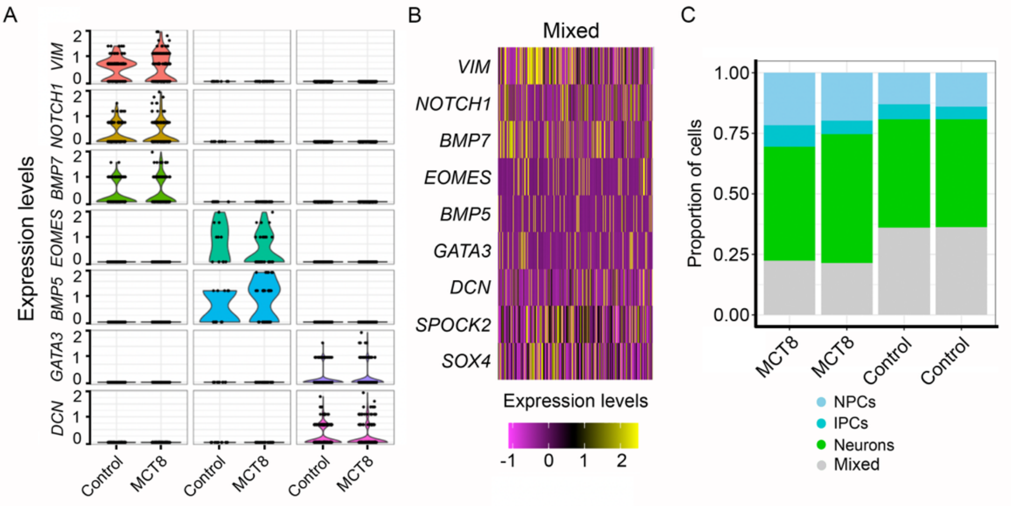

Supplemental Figure 3. A. Spatial transcriptomic violin plots of the expression levels of the indicated markers and the indicated groups of COs. **B**. Heatmap showing the relative

expression levels of the cell identity marker genes in the group of mixed cells. **C**. Histograms of the relative number of cells in the indicated cells and groups of COs.

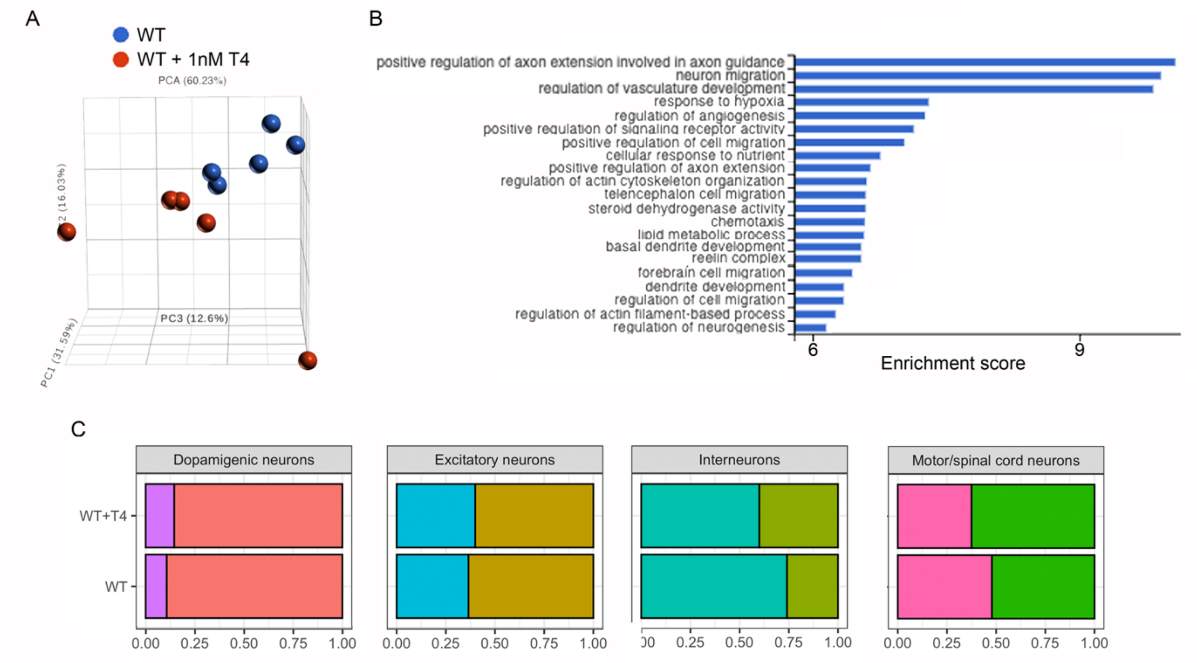

Supplemental Figure 4. A. Principal component plot illustrating differences between control and control + 1 nM T4 NPCs. **B**. Top gene sets with enrichment scores >6. **C**. Histograms of the relative number of cells in the indicated type of neurons.
